# Tipping Growth Inhibition into Apoptosis by Combining Treatment with MDM2 and WIP1 Inhibitors in p53^WT^ Uterine Leiomyosarcoma

**DOI:** 10.3390/cancers14010014

**Published:** 2021-12-21

**Authors:** Victoria Chamberlain, Yvette Drew, John Lunec

**Affiliations:** 1 Newcastle University Centre for Cancer, Newcastle University, Newcastle upon Tyne NE2 4HH, UK; v.m.chamberlain2@ncl.ac.uk (V.C.); yvette.drew@bccancer.bc.ca (Y.D.); 2 BC Cancer Centre Vancouver and Faculty of Medicine, University of British Columbia, Vancouver, BC V5Z 4EH, Canada

**Keywords:** RG7388/HDM201, GSK2830371, WIP1/PPM1D, MDM2, p53, Uterine Leiomyosarcoma, targeted therapy

## Abstract

**Simple Summary:**

Uterine Leiomyosarcoma (uLMS) is a rare (0.8 per 100,000 women), aggressive cancer that predominantly affects post-menopausal women. Prognosis for these women is poor, with relapse following primary treatment occurring in up to 70% of cases. For women with recurrent or advanced uLMS, there is no optimal therapeutic strategy, and research to develop novel, targeted therapies is needed. This study investigates novel combinations in uLMS preclinical models. We present encouraging results using MDM2 inhibitor-based combination treatments, including the WIP1 phosphatase inhibitor GSK2830371. These data suggest that women with uLMS could respond to such combination treatments; therefore, these should be investigated in clinical trials. As these agents do not bind to and interfere with DNA, they offer a non-genotoxic alternative to the cytotoxic chemotherapy currently used in the recurrent setting.

**Abstract:**

As there is no optimal therapeutic strategy defined for women with advanced or recurrent uLMS, there is an urgent need for the discovery of novel, targeted approaches. One such area of interest is the pharmacological inhibition of the MDM2-p53 interaction with small-molecular-weight MDM2 inhibitors. Growth inhibition and cytotoxic assays were used to evaluate uLMS cell line responses to MDM2 inhibitors as single agents and in combination, qRT-PCR to assess transcriptional changes and Caspase-Glo 3/7 assay to detect apoptosis. RG7388 and HDM201 are potent, selective antagonists of the MDM2-p53 interaction that can effectively stabilise and activate p53 in a dose-dependent manner. GSK2830371, a potent and selective WIP1 phosphatase inhibitor, was shown to significantly potentiate the growth inhibitory effects of RG7388 and HDM201, and significantly increase the mRNA expression of p53 transcriptional target genes in a p53^WT^ cell line at a concentration that has no growth inhibitory effects as a single agent. RG7388, HDM201 and GSK2830371 failed to induce apoptosis as single agents; however, a combination treatment tipped cells into apoptosis from senescence. These data present the possibility of MDM2 and WIP1 inhibitor combinations as a potential treatment option for p53^WT^ uLMS patients that warrants further investigation.

## 1. Introduction

One of the main challenges facing clinicians when treating women with recurrent/advanced uLMS, is the limited evidence for guiding systemic treatment options beyond primary debulking surgery. This, coupled with the fact that uLMS is rare, often discovered incidentally during routine hysterectomy for fibroids, and has high rates of relapse means that there is an urgent need to discover effective, targeted therapies. One potential area of interest is the pharmacological inhibition of the ‘MDM2-p53 binding interaction’ with low-molecular-weight MDM2 inhibitors. Several MDM2 inhibitors were developed that showed promise pre-clinically and are currently in early-phase clinical trials, although not specifically in uLMS patients [1,2,3]. If successful, these could provide a non-genotoxic alternative treatment option for p53^WT^ uLMS patients.

The p53 protein is a tumour suppressor that, under normal conditions, is present in either its latent form or maintained by constant turnover at low levels [4]. However, in response to physiological stress, its half-life extends, resulting in the accumulation of p53 in the nucleus where it can act as a transcription factor [5,6]. As such it can bind to and activate the expression of a broad range of target genes, resulting in different cellular responses, including DNA repair, cell cycle arrest, senescence or apoptosis [4,7,8]. As p53 is the most frequently inactivated gene in human cancers, it has been extensively studied, both as a prognostic factor and as a potential therapeutic target for re-activation. The most common mutations are substitutions leading to a reduction or loss of function of p53. Some of the point missense mutations also result in the accumulation of a mutant protein, which, because of an altered conformation, is no longer recognised by its negative regulator, MDM2 [9,10,11].

Under normal physiological conditions, p53 levels are kept low, as it is constantly degraded by MDM2. MDM2 is a multi-functional protein, the main function of which is to act as an E3 ubiquitin ligase that binds to p53 at the N-terminus and ubiquinates several lysine residues in the C-terminal domain, targeting p53 for proteasomal degradation [12,13]. As the structural details of the binding between MDM2 and p53 were elucidated, and the importance of this interaction understood, preventing MDM2 from binding to p53 is actively pursued as a therapeutic strategy. MDM2 has a well-defined, small hydrophobic binding site for three key residues of p53. As such, there is a focus on developing low-molecular-weight compounds that occupy this hydrophobic pocket, with a high affinity and specificity to prevent the binding interaction between p53 and MDM2 [14]. This allows the release and accumulation of WTp53 to carry out its growth inhibitory and/or pro-apoptotic functions.

RG7388 (Idasanutlin) and HDM201 (Siremadlin) are two small-molecular-weight MDM2 inhibitors that both had acceptable safety profiles in early-phase clinical trials. However, MDM2 inhibitor monotherapy may not be sufficient to achieve sustained clinical responses in cancer patients. In addition, resistance to single-agent treatment has emerged, possibly through the positive selection of p53-mutated clones [15]; therefore, combination treatments are being investigated. Combination therapy could improve efficacy and also reduce the risk of developing resistance.

*PPM1D* (Protein Phosphatase, Mg^2+^/Mn^2+^ Dependent, Delta Isoform)*,* an oncogene that is commonly found to be overexpressed or amplified in p53^WT^ cancers, including breast and ovary [16,17,18], encodes the protein wild-type, p53-induced phosphatase 1 (WIP1), which is a direct transcriptional target of p53. However, WIP1 is also involved in the homeostatic regulation of p53 as, following cellular stress, WIP1 binds directly to p53 and dephosphorylates it at serine 15, thereby forming an auto-regulatory negative feedback loop [19]. WIP1 phosphatase activity also has a negative feedback regulatory effect on the upstream kinases that phosphorylated p53. In silico systems analysis modelling indicates that, following p53 activation, WIP1 may have an important role to play, along with MDM2 as an additional negative feedback regulator in the oscillatory behaviour of the p53 signalling network, and thus potentially in deciding cell fate. Modelling indicates that, at the single-cell level, treatment with an MDM2 inhibitor alone induces p53 in an oscillatory manner, resulting in cell cycle arrest, whereas cell death may require a state of sustained p53 activation. However, when MCF7 cells, modified with siRNA to knock down WIP1, were treated with Nutlin-3, the sustained activation of p53 was achieved resulting in a ~90% cell death [20]. GSK2830371 is a small-molecule, allosteric inhibitor of WIP1 that inhibits its enzymatic activity and enhances its ubiquitin-mediated degradation [21]. Pre-clinical studies with a wide range of cell lines have shown in vitro that WIP1 inhibition with GSK2830371 potentiates the effect of MDM2 inhibitors in a p53-dependent manner, at concentrations where, as a single agent, it has limited effects [21,22,23,24,25].

Treatment with RG7388 or HDM201 alone may be limited by subsequent anti-apoptotic mechanisms; therefore, great effort was invested in combination strategies targeting the apoptosis pathway. Evading apoptosis is one of the hallmarks of cancer and can lead to chemotherapy resistance [26]. Therefore, therapeutic approaches that target negative regulators of apoptosis warrant investigation. The use of BH3 mimetics, small-molecule compounds that antagonise the anti-apoptotic BCL2 family proteins, which negatively regulate the intrinsic apoptosis pathway, are one such approach. BCL2, MCL-1 and BCL-X_L_, the main anti-apoptotic proteins, were identified as cellular oncogenes, as they are frequently found to be overexpressed in human cancers and prevent normal or protective cell death mechanisms; therefore, there is a focus on developing inhibitors that have a high affinity and relative specificity for one or more members of this protein family [27,28,29,30].

Venetoclax, formally known as ABT-199, a potent and selective inhibitor of BCL2, gained FDA approval in 2016 for patients with chromosome 17 p deleted Chronic Lymphocytic Leukaemia (CLL). Interestingly, Hoffman-Luca et al. reported that Acute Myeloid Leukaemia (AML) cell lines that displayed resistance to MDM2 inhibitors retained sensitivity to BCL2 inhibitors [31], and Carter et al. reported the synergistic effects of an MDM2 BCL2 inhibitor combination in blast crisis Chronic Myeloid Leukaemia (CML) cells [32]. In 2018, encouraging results were presented from a phase 1b trial investigating RG7388 in combination with Venetoclax in patients with AML. Resistance to the inhibition of BCL2 was overcome by inhibiting MDM2; cancer cells can become resistant to BCL2 inhibition by increasing the production of other anti-apoptotic proteins, such as MCL-1. MCL-1 is selectively targeted for proteasomal degradation, which then facilitates p53-mediated apoptosis, resulting in cell death [33]. More recently, in 2020, Decaudin et al. reported a synergistic combination between ABT263, a BCL2/X_L_/W inhibitor, and HDM201 in uveal melanoma cells and patient-derived xenografts [34].

MCL-1 specific inhibitors, however, proved challenging to design, and drug discovery/development pipelines displayed varying degrees of success, in part due to the large, rigid, hydrophobic, BH3-binding groove of MCL-1 [35]. MIM1, a highly specific low-molecular-weight inhibitor of MCL-1, demonstrated promise in preclinical studies by inducing apoptosis in leukaemia [36], glioblastoma [37], and melanoma cell lines [38]. This study presents novel results from investigations into the potential of GSK2830371, a WIP1 inhibitor; Venetoclax, a BCL2 inhibitor; and MIM1, an MCL-1 inhibitor, to potentiate the effects of MDM2 inhibitors RG7388 and HDM201 in p53^WT^ uLMS cells.

## 2. Materials and Methods

### 2.1. Cell Lines and Reagents

Cell lines were obtained from the American Type Culture Collection (ATCC) and authenticated by short-tandem repeat profiling (NewGene Ltd., Newcastle, UK). MES-SA and SK-UT-1 originate from the uterus and SK-LMS-1 the vulva. Cell lines were routinely cultured in Dulbecco’s Modified Eagle’s Medium (DMEM-D5796) supplemented with 10% (*v*/*v*) foetal calf serum and 1% penicillin-streptomycin (SIGMA). Cell lines were routinely tested for mycoplasma infection. RG7388, HDM201 and Venetoclax were obtained from Selleckchem (Houston, TX, USA); GSK2830371 was purchased from SIGMA; and MIM1 Bio-Techne (Abingdon, UK). All compound stock solutions and serial dilutions were prepared in DMSO (SIGMA) and used in culture media at a final DMSO concentration of 0.5%.

### 2.2. Growth Inhibition Assay

Exponentially growing cells were harvested and seeded in 96-well plates 24 h prior to the addition of either a single agent or combination treatment for 72 h. Plates were fixed with cold Carnoy’s fixative and left overnight at 4 °C to incubate. Plates were then washed with dH_2_O, stained for 30 min with 0.4% (*w*/*v*) Sulforhodamine B (SRB) and dissolved in 1% acetic acid. Plates were then washed 4 times with 1% acetic acid and left overnight to dry. An amount of 10 mM Tris-HCl pH10.5 was used to solubilise the stain and absorbance read at 570 nm. GraphPad PRISM 9 was used to generate GI_50_ values; the concentration at which 50% growth inhibition was achieved. For the dose–response matrix designs, cells were seeded and exposed to a range of drug concentration combinations; the effect on cell numbers after 72 h was determined as described above, then analysed using the SynergyFinder interactive web application to evaluate the response to drug combinations [39].

### 2.3. Clonogenic Assay

Cells were seeded in 6-well plates and left to adhere for 24 h before the drug of interest was added. 72 h later the medium containing drug was removed and replaced with fresh medium, then plates were left to incubate at 37 °C for 14 days to assess colony forming ability. Following incubation, cells were fixed with Carnoy’s fixative and stained using 0.4% crystal violet; colonies were counted using a DOT colony counter (IUL, Barcelona, Spain). Dose-survival curves and LC_50_ concentrations, the lethal concentration at which a 50% reduction in colony forming ability was achieved, were calculated using GraphPad PRISM 9.

### 2.4. Western Immunoblotting

Cells were seeded in 6-well plates at a density of 2.5 × 10^5^/mL in 2 mL supplemented medium and left to adhere for 24 h before the drug of interest was added. After 24 h, medium containing drug was removed and cells washed with 2 mL cold PBS. 40 µL of sodium dodecyl sulfate (SDS) lysis buffer was added to each well and lysates collected. Lysates were heated at 100 °C for 10 min, and then sonicated three times at amplitude 6.0 for 10 s using a MSE Soniprep 150 Plus ultrasonic disintegrator (Henderson Biomedical Ltd., Sydenham, UK). A Pierce™ BCA Protein Assay Kit (ThermoFisher Scientific, Cramlington, UK) was used to quantify the amount of protein in each lysate. From each assay, 35 µg was then loaded onto a 12-well 4–20% Mini-PROTEAN® TGX™ Gel (Bio-Rad, Hertfordshire, UK), the outside wells loaded with SeeBlue™ Pre-stained Protein Standard (Invitrogen, Cramlington, UK) and gel electrophoresis separation of the proteins performed at 180 V for 45 min. The separated proteins were subsequently transferred to a Hybond-C nitrocellulose membrane by orthogonal electrophoresis at 100 V for 30 min. The membrane was then blocked for 1 h in either 5% milk/TBS/Tween or BSA. The membrane was cut into three and each strip incubated in primary antibody overnight at 4 °C. Membranes were washed in TBS/Tween then incubated with horseradish peroxidase (HRP) conjugated secondary antibodies at a 1:1000 dilution for 90 min at room temperature. Membranes were then subject to 4 × 4 min washes in TBS/Tween and imaged using a G:BOX XT4 Chemiluminescence and Fluorescence Imaging System (Syngene, Cambridge, UK), using Clarity Western Enhanced Chemiluminescence (ECL) Substrate (Bio-Rad, Hertfordshire, UK). To remove antibodies, membranes were then incubated in harsh stripping buffer (20% (*v*/*v*) SDS, 12% (*v*/*v*) 0.5 M Tris HCl pH 6.8, 67% (*v*/*v*) ultra-pure water and 1% (*v*/*v*) βeta-mercaptoethanol) in a water bath at 56 °C with agitation for 30 min, before being washed twice with TBS/Tween. For re-probing with different antibodies, the membranes were blocked and the same procedure as mentioned above followed. For details of primary antibodies used see Table A1. Secondary antibodies were from Dako (Copenhagen, Denmark): polyclonal Goat Anti-Rabbit (PO448) and polyclonal Goat Anti-Mouse (PO447). Original western blots are included in Figures S1 and S2.

### 2.5. Quantitative Reverse Transcriptase PCR

An RNeasy Mini Kit (Qiagen 74104) was used in accordance with the manufacturer’s instructions to isolate total RNA, which was then quantified using a NanoDrop ND-1000 Spectrophotometer (ThermoFischer Scientific, Cramlington, UK). A High-Capacity cDNA Reverse Transcription Kit (Applied Biosystems 4368814) was used to convert total RNA to single strand cDNA. To 10 µL Master mix, 1 µg of RNA was added, and the final volume made up to 20 µL with ddH_2_O. Samples were then incubated under the following conditions using a Gene Amp PCR system 2700: 25 °C for 10 min, 37 °C for 120 min hold, 85 °C for 5 s then hold at 4 °C. For the qRT-PCR reaction, SYBR green (Life Technologies, Glasgow, UK), primers (detailed in Table A2), cDNA (10 ng), and ddH_2_O were added to make a final reaction volume of 10 µL, and then the plate was loaded into the QuantStudio 6 Flex Real-Time PCR System (Applied biosystems, Life Technologies, Glasgow, UK). GAPDH was used as the endogenous control and DMSO-vehicle-only-treated cells used as the reference calibrator. RQ values were calculated using the formula 2^ΔΔ^^Ct^, where RQ expresses the fold change relative to the calibrator (DMSO).

### 2.6. Caspase-Glo® 3/7 Assay

Cells were seeded in white 96-well plates (Greiner bio-one 655083) and left to adhere for 24 h. Medium was removed and replaced with 50 µL medium containing drug, and cells were incubated for 24 h. Plates were then left to cool at room temperature for 10 min before adding 50 µL Caspase-Glo 3/7 reagent (1:1 ratio). Plates were protected from light and left to incubate for 30 min, before luminescence was measured on a FLUOstar Omega plate reader. All values were expressed as fold changes relative to DMSO treated control.

### 2.7. Statistical Analysis

Data are presented as the mean ± standard error mean (SEM) of three independent repeats unless otherwise stated. All statistical analyses were conducted using GraphPad PRISM 9 with a *p*-value ≤ 0.05 considered significant.

## 3. Results

### 3.1. RG7388 and HDM201 Are Potent Inhibitors in p53^WT^ uLMS Cells but Not p53^MUT^

**Figure 1:**
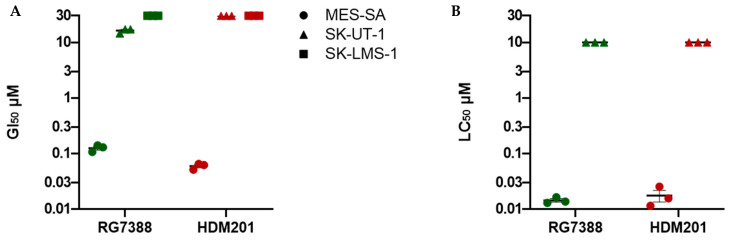
Summary values from growth inhibition and cytotoxicity assays for LMS cell lines treated for 72 h with MDM2 inhibitors, RG7388 and HDM201. (**A**) SRB assay was used to determine GI_50_ values. For SK-UT-1 treated with HDM201, and SK-LMS-1 treated with both RG7388 and HDM201, no GI_50_ value was reached up to the maximum tested dose of 30 µM; (**B)** LC_50_ values generated from clonogenic survival assays for MES-SA and SK-UT-1 against RG7388 and HDM201. For SK-UT-1, no LC_50_ was reached up to the maximum tested dose of 10 µM. Data represent three independent repeats with mean ± SEM.

The p53^WT^ cell line MES-SA exhibited a dose-dependent response to inhibition with RG7388 and HDM201, with GI_50_ values in the nanomolar range, 126 ± 10.07 nM and 60 ± 4.62 nM, respectively (Figure 1A). MES-SA also displayed a reduction in clonogenic cell survival in response to inhibition with RG7388 and HDM201, with LC_50_ values also in the nanomolar range: 14.0 ± 1.0 nM and 18.0 ± 4.2 nM, respectively (Figure 1B). HDM201 was significantly more potent than RG7388 in growth inhibition assays—*p* = 0.0037 (unpaired *t*-test)—but not in clonogenic survival assays, as no significant difference between the two inhibitors was found. Both p53^MUT^ cell lines, SK-UT-1 and SK-LMS-1, were resistant to RG7388 up to 10 µM and HDM201 up to 30 µM. Interestingly, SK-UT-1 showed a sensitivity towards RG7388 at the highest dose of 30 µM, with a GI_50_ value of 16.3 ± 0.9 µM, while SK-LMS-1 remained resistant, indicating that HDM201 had a higher specificity than RG7388 for its MDM2 target, and thus may be less likely to cause off-target effects in patients. However, in clonogenic survival assays, SK-UT-1 displayed resistance to both RG7388 and HDM201 of up to 10 µM, suggesting that even if proliferation is temporarily impaired the replicative potential is not, and that given time, cells are able to recover and continue to proliferate.

### 3.2. GSK2830371 Has No Growth Inhibitory Effects as a Single Agent

**Figure 2:**
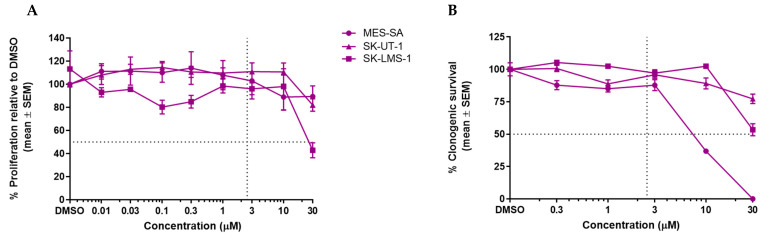
Growth inhibitory and cytotoxic effects of GSK2830371 as a single agent on LMS cell lines. Dashed lines highlight 2.5 µM and 50% proliferation/clonogenic survival. (**A**) Dose-response curves following 72 h incubation with GSK2830371 (SRB assay); (**B**) dose-response curves from clonogenic survival assays used to determine LC_50_ concentrations. Cells were treated for 72 h then left to incubate for 14 days. Data points represent the mean ± SEM of three independent repeats.

No or minimal growth inhibition was observed for doses ≤ 10 µM in all cell lines; however, for SK-LMS-1 at 30 µM there was evidence of a growth inhibition with a GI_50_ value of 27.7 ± 1.6 µM (Figure 2A). Clonogenic survival assays also revealed no evidence of cytotoxicity in all cell lines up to doses of 3 µM. For both p53^MUT^ SK-UT-1 and SK-LMS-1 cell lines, there was slight inhibition at 10 and 30 µM, respectively; but for p53^WT^ MES-SA, there was a dose-dependent inhibition by GSK2830371, with an LC_50_ value of 8.2 ± 0.4 µM (Figure 2B). A sub-growth-inhibitory dose of 2.5 µM GSK2830371 was chosen for the initial evaluation of combination studies with MDM2 inhibitors.

### 3.3. WIP1 Inhibitor GSK2830371 Potentiates the Growth Inhibitory and Cytotoxic Effects of MDM2 Inhibitors on uLMS p53^WT^ Cells

**Figure 3:**
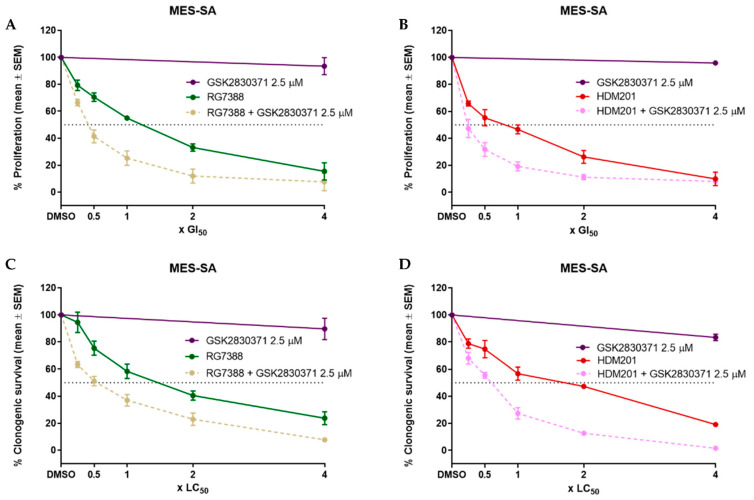

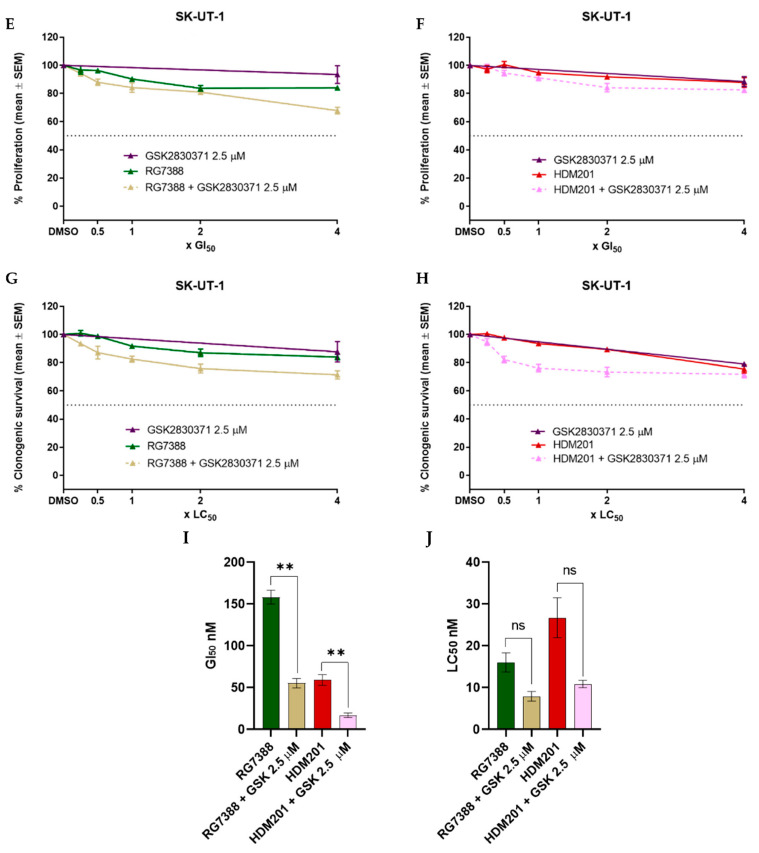
Effects of MDM2 inhibitors in combination with 2.5 µM GSK2830371 on cell proliferation and clonogenic survival. Percentage proliferation or clonogenic survival are expressed relative to DMSO-treated control. Growth inhibition curves for MES-SA cells treated with 2.5 µM GSK2830371 and (**A**) RG7388; (**B**) HDM201; Dose–response curves following clonogenic survival assays for MES-SA cells treated with 2.5 µM GSK2830371 and (**C**) RG7388; (**D**) HDM201; (**E**–**H**) the same as (**A**–**D**) but for p53 mutant SK-UT-1 cells; (**I**) GI_50_ values for MES-SA when treated with single-agent MDM2 inhibitor or combination with 2.5 µM GSK2830371 as determined from growth inhibition assays in (**A**,**B**); (**J**) LC_50_ values for MES-SA when treated with single-agent MDM2 inhibitor or combination with 2.5 µM GSK2830371, as determined from clonogenic survival assays in (**C**,**D**). Statistical significance was evaluated using a paired *t*-test, ** = *p* < 0.01. GSK, GSK2830371; ns, not significant.

GSK2830371, at a dose of 2.5 µM, which had no effect on proliferation, was found to significantly potentiate growth inhibition when combined with RG7388 (Figure 3A) and HDM201 (Figure 3B); GI_50_ values were significantly reduced in combination treatments, *p* < 0.05 as determined by a paired *t*-test, (Figure 3I). Clonogenic assays were then performed to assess the capability of GSK2830371 to potentiate the cytotoxic effects of RG7388 and HDM201. Whilst colony formation was inhibited following a combination treatment with RG7388 (Figure 3C), and HDM201 (Figure 3D), the reduction in LC_50_ values was not significant: *p* = 0.08 and *p* = 0.06, respectively (Figure 3J). The p53^MUT^ SK-UT-1 cells, previously shown to be resistant to RG7388 or HDM201 as single agents, demonstrated only a small combination effect in both growth inhibitions (Figure 3E,F) and clonogenic assays (Figure 3G,H).

**Figure 4:**
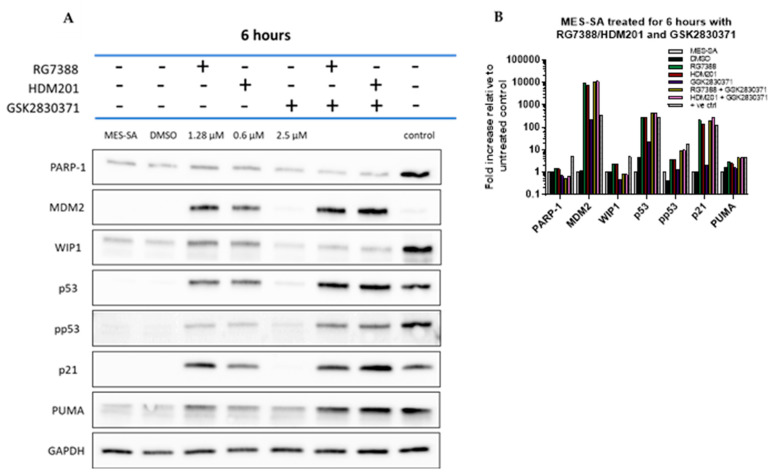
(**A**) Western immunoblot of MES-SA cells treated for 6 h with RG7388, HDM201 and GSK2830371. The positive control was SH5Y5Y cells collected 2 h post 4 Gy X-irradiation (provided by A. Yagbasan). GAPDH was used as the loading control. Doses of RG7388 and HDM201 represent 10 × their GI_50_ concentrations. All strips were from the same membrane which was cut into three. The top strip was probed for WIP1, MDM2 and PARP-1; the second for pp53, p53 and GAPDH; and the third PUMA and p21. (**B**) Densitometry, with values background corrected, normalised to GAPDH, then fold change expressed relative to untreated control.

Western blot analysis (Figure 4) was used to investigate the effect of combination treatment on the p53 molecular signalling pathway. MES-SA cells were treated for 6 h with RG7388 or HDM201 at 10 × their respective GI_50_ concentrations, GSK2830371 at 2.5 µM, or a combination. As single agents, both MDM2 inhibitors induced p53 stabilisation and the upregulation of p21 (p21^WAF1^, CDKN1A); therefore, confirming the functional activation of wild-type p53. GSK2830371 as a single agent did not induce p53 stabilisation. Combination treatment, however, led to an increased p53 stabilisation with increased levels of the p53 transcriptional targets, MDM2 and p21. An increase in phosphorylated p53 (pp53) was also observed with combination treatment and this correlated with the decrease in levels of WIP1, which, when present, acted to dephosphorylate and negatively regulate pp53. Upon inhibiting WIP1, there was also a decrease in the levels of full-length PARP-1 and an increase in PUMA, both suggesting that apoptotic signals increased compared to the single-agent treatment.

### 3.4. Strong Synergy Observed with MDM2 Inhibitors in Combination with GSK2830371

**Figure 5:**
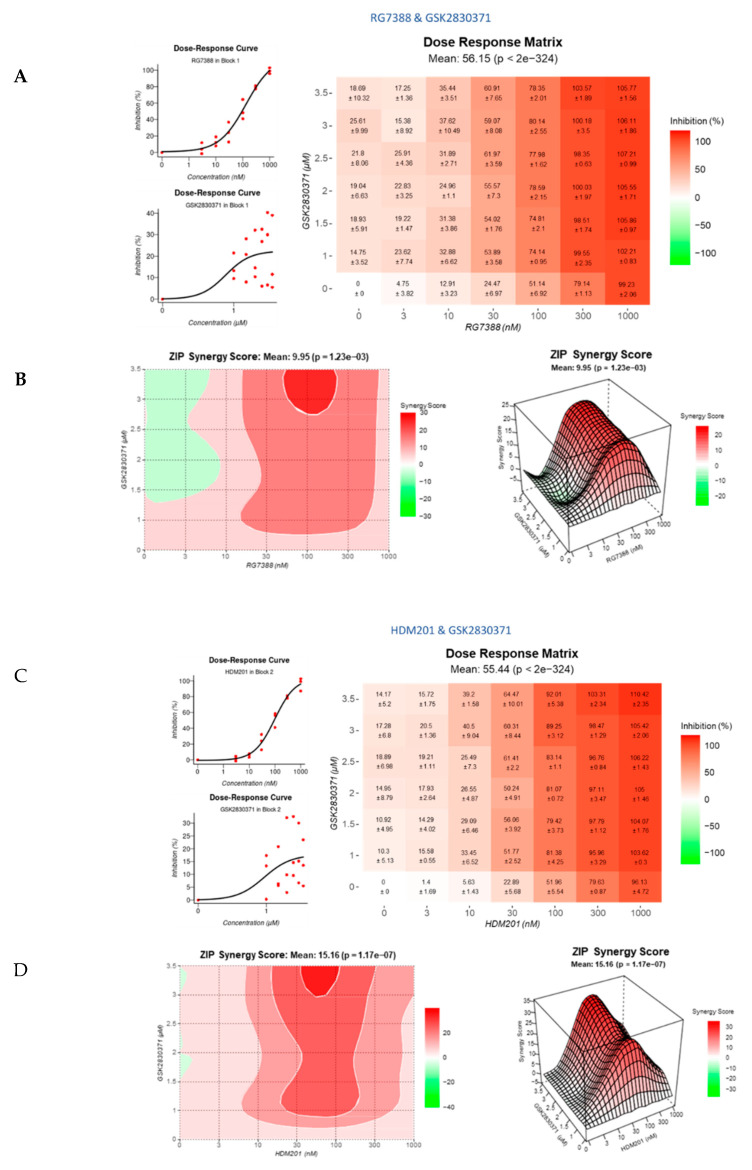
Synergy plots for MDM2/WIP1 inhibitor combination. A dose–response matrix design was used to assess synergy and determine doses at which the greatest synergy was observed, as determined by the Zero Interaction Potency (ZIP) Model. MES-SA cells were treated with either RG7388 or HDM201 and 2.5 µM GSK2830371 for 72 h then growth inhibition was assessed using SRB assay. Dose–response curves, dose–response matrix and synergy plots for RG7388 and GSK2830371 (**A**,**B**), and for HDM201 and GSK2830371 (**C**,**D**).

A dose–response matrix design was used to explore a wider range of combination concentrations to establish the dose of RG7388 or HDM201 that caused the greatest synergy when used in combination with GSK2830371, as determined by the Zero Interaction Potency (ZIP) Model. Both MDM2 inhibitor combinations were considered synergistic with overall synergy scores > 10. For both combinations, RG7388 + GSK2830371 (Figure 5A,B) and HDM201 + GSK2830371 (Figure 5C,D), there was a statistically significant difference between the overall and highest synergy scores recorded (14.63 ± 0.12 vs. 23.45 ± 2.52 for RG7388 + GSK2830371 *p* = 0.0188 and 16.37 ± 1.35 vs. 27.64 ± 1.25 for HDM201 + GSK2830371 *p* = 0.0079), highlighting the importance of optimal dose selection for combination studies. The strongly synergistic effect with GSK2830371 was observed to the same extent for both MDM2 inhibitors, suggesting that there was an in-class drug effect. Heatmaps, generated by SynergyFinder, indicated that, for both combinations, the area where the greatest synergy was observed was between 30–100 nM, for either MDM2 inhibitor, and 2.5–3.5 µM for GSK2830371, where 80–90% inhibition was achieved. Importantly, these results are seen in doses of the MDM2 inhibitor that can be achieved clinically [40].

### 3.5. Combination with GSK2830371 Induces Irreversible Growth Arrest Following 72 h Treatment of p53^WT^ uLMS Cells with nM Concentrations of MDM2 Inhibitors

**Figure 6:**
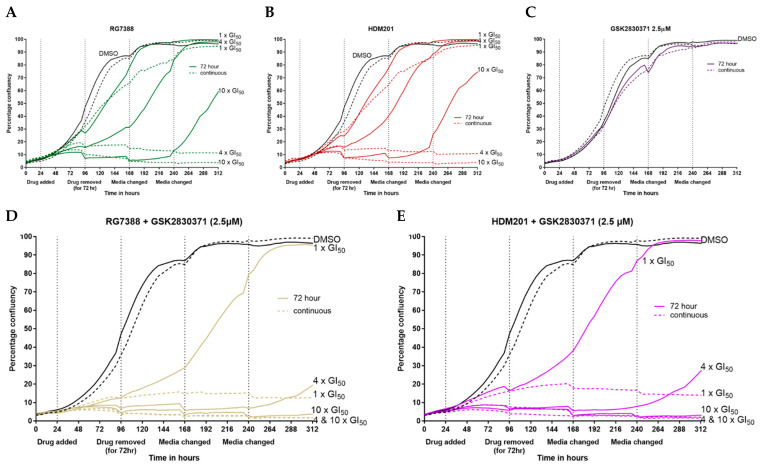

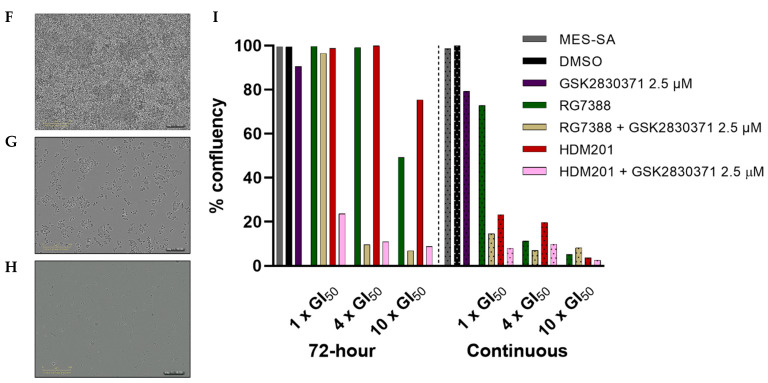
Images and confluency data captured using the IncuCyte incubator camera system. MES-SA cells were treated with drug for either 72 h or continually dosed for 288 h. Medium was replaced every 72 h and phase contrast images taken every 4 h. Growth curves for (**A**) RG7388; (**B**) HDM201; (**C**) GSK2830371; (**D**) RG7388 + GSK2830371; (**E**) HDM201 + GSK2830371, with solid lines representing 72 h treatment and dashed lines continual treatment; representative phase contrast images taken on day 11 at 18:00 h for (**F**) DMSO treatment for 72 h; (**G**) RG7388 at 10 × GI_50_ concentration for 72 h; (**H**) RG7388 at 10 × GI_50_ + 2.5 µM GSK2830371 treatment for 72 h; (**I**) percentage confluency of each well following either 72 h or continual treatment with 1, 4 or 10 × GI_50_ concentrations of RG7388 or HDM201, alone or in combination with 2.5 µM GSK2830371. Percentage confluency of each well was calculated using an integrated confluence algorithm and reflects both cell number and area of the well covered by cells.

The IncuCyte incubator camera system was used to capture phase contrast images every 4 h to accurately determine confluency following either 72 h treatment or continual exposure for 288 h. The medium was replaced every 72 h. MES-SA cells recovered their ability to proliferate following exposure to RG7388 (Figure 6A) or HDM201 (Figure 6B) for 72 h at 1, 4 and 10 × GI_50_ concentrations, although growth was retarded and delayed. However, when MES-SA cells were continually dosed with either RG7388 (Figure 6A) or HDM201 (Figure 6B) they were unable to regrow at any dose, the only exception being RG7388 at 1 × GI_50_ concentration. A 72 h treatment with 2.5 µM GSK2830371 had little to no effect on the overall proliferation but did initially slow cell growth (Figure 6C). A similar effect was observed when cells were continuously dosed with GSK2830371, only to a greater extent when ~80% overall confluency was reached (Figure 6C). In marked contrast to the reversible growth inhibition with MDM2 inhibitor alone, when treated for 72 h with either RG7388 (Figure 6D) or HDM201 (Figure 6E) in combination with 2.5 µM GSK2830371, cells were unable to regrow again at any dose, with only one exception: RG7388 + GSK2830371 at 1 × GI_50_ dose of RG7388. These results highlight the potential of this treatment combination, as the addition of 2.5 µM GSK2830371 to low doses of MDM2 inhibitors, 4 × GI_50_, can generate the same growth inhibitory effects following 72 h treatment that can only be achieved through continual dosing with the MDM2 inhibitors as single agents.

### 3.6. Combined MDM2 and WIP1 Inhibition Significantly Increases Transcript Levels of Pro-Apoptotic Genes in p53^WT^ uLMS Cells

**Figure 7:**
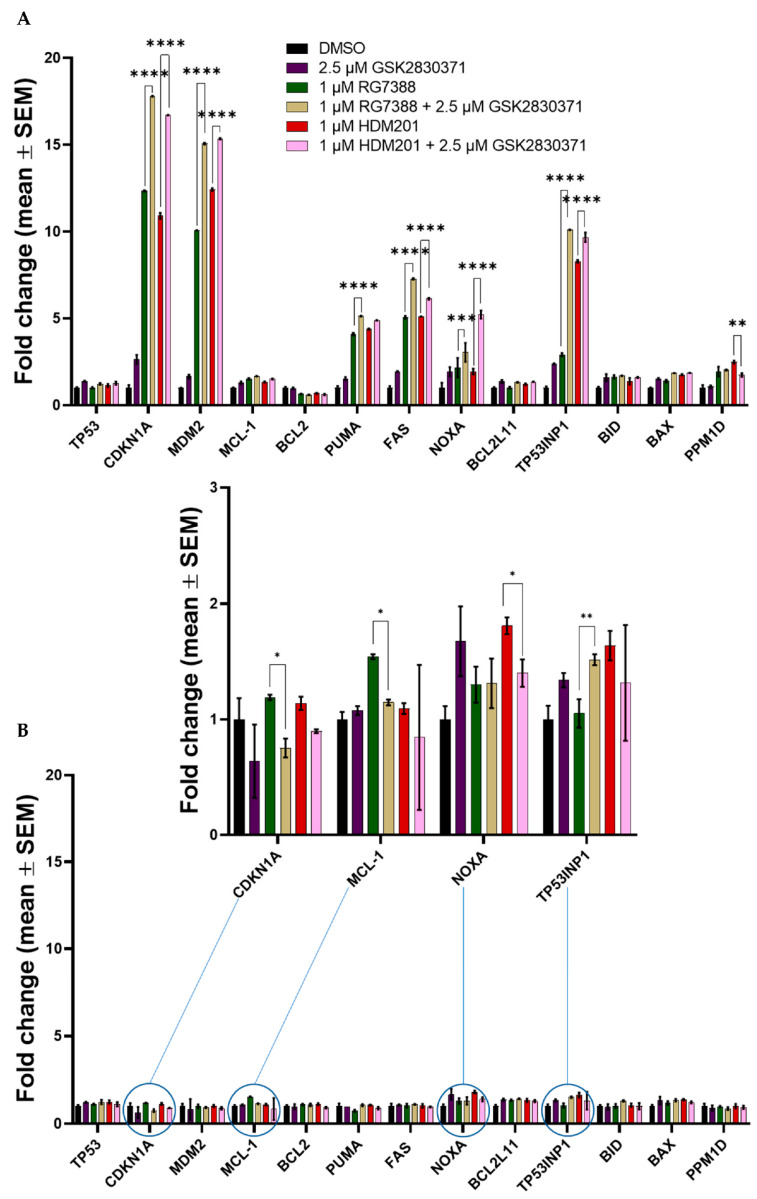
Fold change in mRNA expression of selected p53 transcriptional target genes following 6 h treatment with either single agent RG7388, HDM201, 2.5 µM GSK2830371 or a combination in (**A**) MES-SA; (**B**) SK-UT-1. GAPDH was used as the endogenous control and DMSO-treated cells were used as the calibrator between repeats. RQ values were calculated using the formula 2^ΔΔ^^Ct^. Bars represent the mean ± SEM. Statistical significance was determined by two-way ANOVA with Tukey’s post hoc test for multiple comparisons, significance taken at *p* < 0.005. Only significant changes between single-agent MDM2 inhibitor treatment and the combination with 2.5 µM GSK2830371 are displayed on the graphs. All others are listed in Table A3 for MES-SA and A4 for SK-UT-1. * *p* ≤ 0.05, ** *p* ≤ 0.01, *** *p* ≤ 0.001, **** *p* ≤ 0.0001.

To determine if combination treatment also enhanced the transcriptional activity of p53, qRT-PCR was used to investigate the mRNA expression levels of selected p53 transcriptional target genes involved in cell cycle arrest and apoptosis. MES-SA and SK-UT-1 were treated with 1 µM RG7388 or 1 µM HDM201 alone or in combination with 2.5 µM GSK2830371 for 6 h. All statistically significant fold changes, as determined by a 2-way ANOVA with Tukey’s post hoc test, are listed in Table A3 for MES-SA and Table A4 for SK-UT-1; Figure 7 only displays those between RG7388 and HDM201 single-agent treatments and the combination with 2.5 µM GSK2830371. Consistent with their p53 mutational status, fold changes observed in SK-UT-1, p53^MUT^, (Figure 7B) were much lower than in MES-SA, p53^WT^ (Figure 7A). For both MES-SA and SK-UT-1 there was little to no change in the transcript levels of *TP53* itself, or of the anti-apoptotic gene *BCL2* following any treatment. For MES-SA, *BCL2L11* also remained unchanged, as did *MDM2*, *PUMA*, *FAS*, *BID,* and *PPM1D* for SK-UT-1. Generally, there was clear induction of p53 target genes following the treatment with either RG7388 or HDM201, which was further increased with the addition of 2.5 µM GSK2830371 in MES-SA, but not SK-UT-1.

For MES-SA, *CDKN1A* (a gene involved in cell cycle control, the inhibition of proliferation and implicated in senescence), *MDM2,* and pro-apoptotic genes, *FAS*, *NOXA*, and *TP53INP1* were all induced to significantly higher levels following combination treatment with GSK2830371, compared to treatment with either MDM2 inhibitor alone. Surprisingly, *PPM1D* was the only gene whose expression was significantly reduced following combination treatment with 2.5 µM GSK2830371 compared to HDM201 single-agent treatment. Transcript levels of the *PUMA* pro-apoptotic gene were also increased following combination treatment; however, the difference was only significant for RG7388 and GSK2830371. *BID, BCL2L11,* and *BAX,* pro-apoptotic genes involved in the initiation and regulation of the intrinsic apoptosis pathway, showed only a modest induction compared with other p53 target genes, with no significant difference between any of the treatment options. These results are consistent with previous studies showing either no or modest induction of *BAX* following treatment with MDM2 inhibitors (RG7388 and Nutlin-3a) in a panel of ovarian or cutaneous melanoma cell lines, respectively [25,41]. Interestingly, treatment with 2.5 µM GSK2830371 alone significantly induced some of the p53 transcriptional target genes: *CDKN1A, MDM2, FAS, NOXA, TP53BP1* and *BID,* although the induction was relatively small with none greater than 3-fold. When considered alongside the earlier described results showing that 2.5 µM GSK2830371 treatment alone had little to no effect on cell proliferation, this indicates that such small changes are below the threshold required for a functional impact.

For the p53^MUT^ SK-UT-1 cells, any changes in expression were modest, with no greater than a 2-fold induction over DMSO control. However, it was interesting to note that the statistical differences found between single-agent MDM2 treatment and combination treatment with 2.5 µM GSK2830371 were reductions in transcriptional activity, except for *TP53INP1.*

### 3.7. GSK2830371 Combination with MDM2 Inhibitors Induces Apoptosis

**Figure 8:**
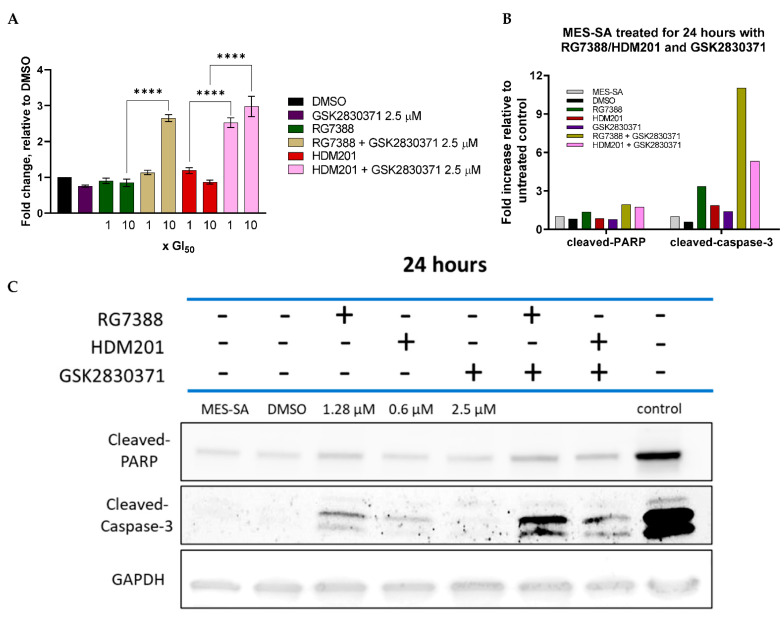
(**A**) Caspase-3/7 catalytic activity in MES-SA cells following 24 h treatment with either RG7388, HDM201 or GSK2830371 as single agents and in combination. Data are expressed as the mean fold change relative to DMSO-treated control, calculated from three independent repeats. Statistical significance was evaluated by a one-way ANOVA with Tukey’s post hoc test for multiple comparisons. **** *p* ≤ 0.0001. (**B**) Western Immunoblot of MES-SA cells treated for 24 h with RG7388, HDM201 and GSK2830371. Positive control was SH5Y5Y cells collected 4 h post X-irradiation with 2 Gy (provided by A. Yagbassan). Doses of RG7388 and HDM201 represent 10 × their GI_50_ concentrations. All strips were from the same membranes, which were cut into three. The top strip was probed for cleaved-PARP, the middle GAPDH, and the bottom cleaved-caspase-3. (**C**) Densitometry. Values were corrected for background signal, normalised to GAPDH, and then fold change was expressed relative to untreated control. The positive control was not included in the densitometry plot.

As the transcriptional activity of several pro-apoptotic genes was significantly enhanced in p53^WT^ cells following combination treatment, the catalytic activity levels of caspases 3 and 7 were assessed to test whether this resulted in increased caspase-3/7-dependent apoptosis. MES-SA cells were treated for 24 h with either single-agent MDM2 inhibitor at 1 or 10 × GI_50_ concentrations (10× only for Western blot analysis), single-agent GSK2830371 at 2.5 µM or a combination of both (Figure 8), then the levels of activity for caspases 3 and 7 were determined using a Caspase-Glo-3/7 assay (Figure 8A), or levels of cleaved-PARP and cleaved-caspase-3 tested using Western immunoblotting (Figure 8B,C). Neither of the MDM2 inhibitors showed evidence of induced caspase-3/7 activity at 1 or 10 × GI_50_ concentrations, nor did 2.5 µM GSK2830371 alone. Interestingly, however, combination treatment led to a significant increase in the levels of caspase-3/7 activity detected with HDM201 at either 1 or 10 × GI_50_ concentrations (*p <* 0.001) but only at 10× for RG7388 (*p* < 0.0001). Western blot analysis showed an increase in both cleaved-PARP and cleaved-caspase-3 following combination treatment, when compared to single-agent treatment alone, indicating that the apoptosis induced by combination treatment is caspase dependent, supporting the caspase-3/7 catalytic activity data.

### 3.8. BCL2 and MCL-1 Inhibitors in Combination with MDM2 Inhibitors Fail to Lower the Apoptotic Threshold Required to Tip p53^WT^ Cells into Apoptosis

**Figure 9:**
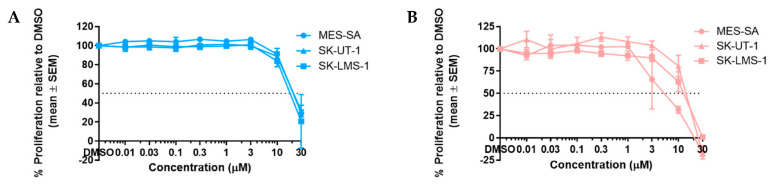
Growth inhibition curves for MES-SA, SK-UT-1 and SK-LMS-1 following incubation for 72 h with (**A**) Venetoclax; (**B**) MIM1 as determined by SRB. Data points represent mean ± SEM of three independent repeats.

Since the WIP1 inhibitor experiments demonstrated that MES-SA could be induced to undergo caspase-3/7 dependent apoptosis, Venetoclax, a specific BCL2 inhibitor, and MIM1, a specific MCL-1 inhibitor, were used to investigate whether inhibiting MDM2, in combination with either BCL2 or MCL-1, would also promote apoptosis. The hypothesis being that the inhibition of either BCL2 or MCL-1, anti-apoptotic proteins, would lower the apoptotic threshold so that MES-SA cells would be pushed into apoptosis rather than growth arrest. MES-SA, SK-UT-1 and SK-LMS-1 were resistant to inhibition with single-agent Venetoclax up to 10 µM (Figure 9A) and MIM1 up to 3 µM (Figure 9B), as determined by SRB. MES-SA, SK-UT-1 and SK-LMS-1 displayed a similar response to Venetoclax, but the response to MIM1 varied; MES-SA was the most sensitive with SK-LMS-1 the least.

**Figure 10:**
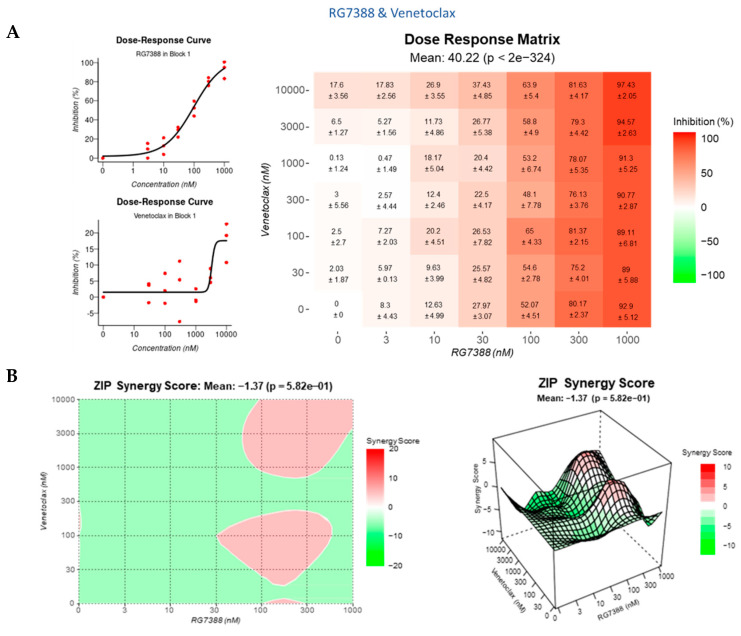

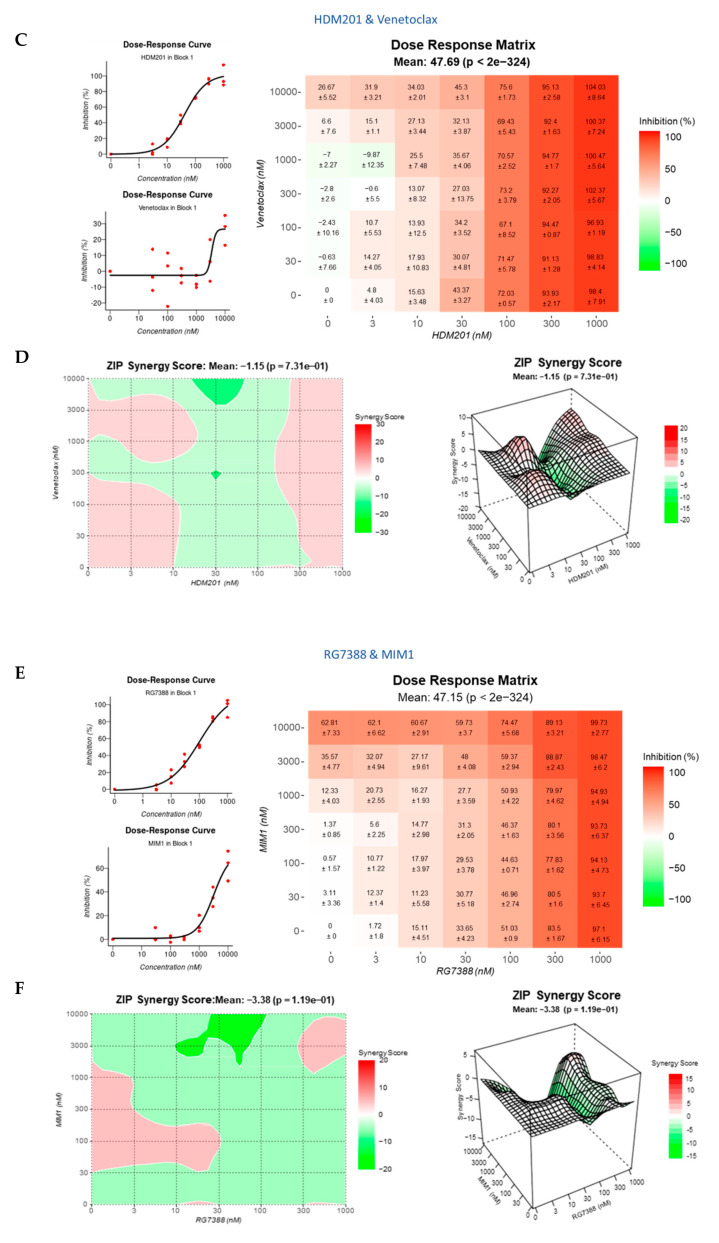

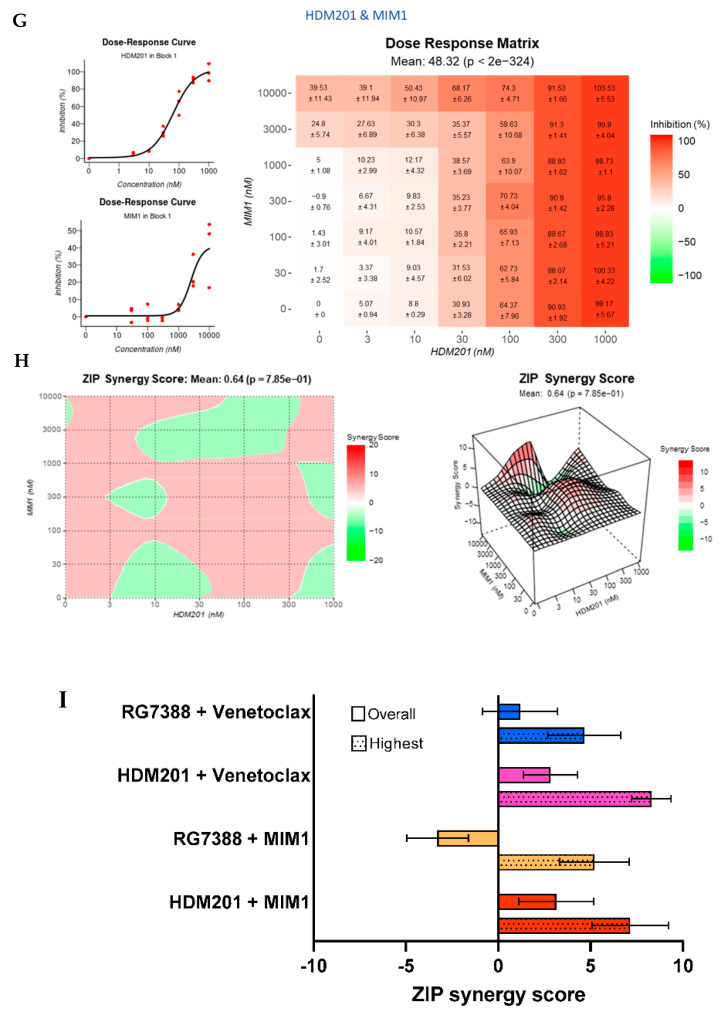
Dose–response curves for single agents, dose–response matrices (inhibition) and synergy maps generated using SynergyFinder for (**A**,**B**) Venetoclax and RG7388; (**C**,**D**) Venetoclax and HDM201; (**E**,**F**) MIM1 and RG7388; and (**G**,**H**) MIM1 and HDM201. On the synergy maps the areas with the greatest synergy are marked with a square; (**I**) Summary plot displaying the overall and highest synergy scores for each combination as determined by the Zero Interaction Potency (ZIP) Model. MES-SA cells were treated for 72 h and growth inhibition was assessed using the SRB assay. Results were then analysed using SynergyFinder. Data represent three independent repeats with mean ± SEM.

A dose–response matrix design was used to evaluate potential synergy for the combinations: RG7388 + Venetoclax (Figure 10A,B), HDM201 + Venetoclax (Figure 10C,D), RG7388 + MIM1 (Figure 10E,F) and HDM201 + MIM1 (Figure 10G,H). All four combinations resulted in an additive effect, with limited the overall evidence of synergy, using the ZIP model. Overall synergy scores ranged from −3 to + 3 with peak synergy scores all still lower than 10 (Figure 10I).

**Figure 11:**
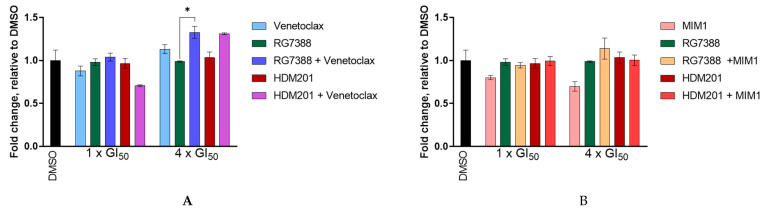
Caspase-3/7 catalytic activity in MES-SA cells following 24 h treatment with (**A**) Venetoclax and or RG7388/HDM201; (**B**) MIM1 and or RG7388/HDM201, assessed by Caspase-Glo 3/7 assay. Data are presented as the mean fold change relative to DMSO control, calculated from three independent repeats. Statistical significance was evaluated by one-way ANOVA with Tukey’s post hoc test for multiple comparisons, * *p* ≤ 0.05.

Even though the combination treatment failed to induce synergy, the levels of caspases 3 and 7 were assessed, as the main aim was to test the hypothesis that the inhibition of these anti-apoptotic proteins could tip the balance of pro-apoptotic and anti-apoptotic signals to induce apoptosis. MES-SA cells were treated for 24 h with either single-agent MDM2 inhibitor at 1 or 4 × GI_50_ concentrations, single-agent BCL2 inhibitor, Venetoclax at 1 or 10 µM, or a combination of both (Figure 11A). Doses of 1 and 10 µM were used for Venetoclax, as no GI_50_ value was obtained for Venetoclax as a single agent. There was little to no increase detected in the levels of caspase 3 or 7, with the only statistically significant difference noted between RG7388 and RG7388 (4 × GI_50_) + Venetoclax (10 µM), *p* = 0.0143. However, even though statistically significant (*p* = 0.0143), the fold increase was less than 1.5.

It was further hypothesised that MDM2 inhibition, in combination with MCL-1 inhibition, would lower the apoptotic threshold in MES-SA and drive the cells into apoptosis. However, this was not supported by the results presented in Figure 11B. When MES-SA cells were treated with either single-agent MDM2 inhibitors at 1 or 4 × GI_50_ concentrations, single agent MCL-1 inhibitor, MIM1 at 1 or 10 µM, or a combination of both, there was no statistically significant difference in fold change, relative to DMSO control for any comparison. Similar to Venetoclax, no GI_50_ value was calculated for MIM1 as a single agent at physiologically relevant doses; therefore, 1 and 10 µM doses were used. Whilst Venetoclax and MIM1 may be able to induce apoptosis in other cell types, they were unable to do so with the uLMS cell lines.

## 4. Discussion

RG7388 and HDM201 are potent, selective antagonists of the MDM2-p53 interaction that can effectively stabilise and activate p53 in a dose-dependent manner. Following treatment with MDM2 inhibitors, two distinct responses can occur: either cell cycle arrest (quiescence or senescence) or apoptosis [10,11]. Following cellular stress, one of the main functions of activated p53 is to trigger cell-cycle arrest through the induction of the downstream transcriptional target p21^WAF1^ (CDKN1A), which encodes a cyclin-dependent kinase inhibitor and arrests cells in the G_1_ and G_2_ phases [42]. It is thought that cell cycle arrest is prioritised over the induction of apoptosis as a means of protection; the priority is to stop the propagation of cells with damaged DNA, with apoptosis being the ultimate end point. Therefore, it was suggested that apoptosis could only occur under certain conditions that may be dependent on cell type, once a particular transcriptional threshold is achieved through either p53 reactivation or suppression of its negative regulators [43,44,45,46]. The main aim of this research was to identify targeted combination treatments capable of inducing apoptosis at lower threshold doses.

Whilst the overexpression of MDM2 was linked to chemoresistance, it was also reported that, in response to MDM2 inhibition, it is these cells that preferentially undergo apoptosis [47,48,49,50,51,52]. Genomic data profiled from 207 soft-tissue sarcoma patients revealed that the most common copy number alteration was the amplification of MDM2 that occurred in 56 (27.1%) of the samples [53]; however, when considering just uterine sarcomas, MDM2 was only found to be amplified in 6/108 (5.6%) and when narrowing this down further to specifically uLMS, in only 3/80 (3.8%) [54], suggesting that amplification of MDM2 in uLMS patients is not a common event. Therefore, MDM2 inhibitor treatment alone may not induce apoptosis in this subset of sarcoma patients, and might instead result in transient cell cycle arrest.

As well as MDM2, the PPM1D/WIP1 phosphatase has an additional negative auto regulatory effect on p53, by dephosphorylating the key amino acid residues required for its activation, and was implicated in resistance mechanisms to p53-dependent therapies. In the current research project, GSK2830371, a potent and selective WIP1 phosphatase inhibitor, displayed minimal growth inhibitory effects on LMS cell lines as a single agent at doses of up to 10 µM, irrespective of p53 status, consistent with previous reports across a wide range of *PPM1D* (WIP1) non-amplified and non-mutant cell lines [23,25,55]. However, Gilmartin et al. and Esfandiari et al. reported that, in cells with amplified *PPM1D* or activated by mutation, GSK2830371 inhibited cell growth with IC_50_ values < 4 µM, but not in WIP1-amplified p53^MUT^ cells (e.g., BT474) [21,23].

It was previously reported that GSK2830371 potentiated the effect of MDM2 inhibition in p53^WT^ cell line [23,25,55]; hence, it was hypothesised that GSK2830371 would also potentiate the effect of MDM2 inhibitors on p53^WT^ uLMS cells. Consistent with previous results, GSK2830371 was shown to significantly potentiate the growth inhibitory effects of RG7388 and HDM201 at 2.5 µM (Figure 3A–D), and significantly increase the mRNA expression of p53 transcriptional target genes (Figure 7A).

To confirm that the potentiation observed with either RG7388 or HDM201 and GSK2830371 was p53 dependent, p53^MUT^ SK-UT-1 uLMS cells were also tested; unlike the p53^WT^ MES-SA cells, no potentiation was observed following combination treatment (Figure 3E–H). Interestingly, there was a small but statistically significant increase in the expressions of some of the p53 transcriptional target genes; *CDKN1A, MCL-1, NOXA* and *TP53INP1*; although, all fold increases were less than 2, and as displayed in Figure 3E–H, were insufficient for affecting cell growth, confirming that the potentiation observed was p53 dependent. A similar finding was reported by Wu et al. in the p53^MUT^ WM35-R cutaneous melanoma cell line [25].

The cellular outcome of the response to signalling through the p53 network depends on the strength of upstream and auto-regulatory signals to p53 and the balance of downstream pro-apoptotic and anti-apoptotic proteins. Our results show that amplifying the signalling to p53, by inhibiting WIP1 phosphatase and enhancing p53 activation by MDM2 inhibitors, was more effective for pushing cells into apoptosis than inhibiting the anti-apoptotic proteins BCL2 and MCL-1, which acted downstream of p53. As single agents, neither the MDM2 inhibitor nor GSK2830371 increased caspase 3/7 activity. However, there was a significant increase in caspase-3/7 activity (Figure 8A) following treatment with either MDM2 inhibitor in combination with GSK2830371, as well as an increase in both cleaved-PARP and cleaved-caspase-3 (Figure 8C), suggesting that MDM2 inhibitor treatment alone caused the insufficient activation of p53 and/or suppression of its negative regulators at the doses tested, triggering apoptosis in MES-SA uLMS cells. Figure 7 shows that, following combination treatment, there was a significant increase in the transcript levels of *CDKN1A,* indicative of increased p53 activation, coupled with a significant increase in the mRNA expression of pro-apoptotic genes—*PUMA* (previously shown to be pivotal in deciding cell fate in response to MDM2 inhibitors) *FAS, NOXA* and *TP53INP1*—indicating a sustained increase in the activation of p53 transcriptional activity consistent with the observed synergy and push of the cells into apoptosis [46]. Figure 4A shows that the increase in transcript levels of *PUMA* translated to an increase in protein levels following combination treatment.

As BCL2 and MCL-1 are considered two of the main anti-apoptotic proteins, it was hypothesised that, by combining MDM2 inhibitors with either Venetoclax (BCL2 inhibitor) or MIM1 (MCL-1 inhibitor), the apoptotic threshold could be lowered, and thus cells would undergo apoptosis. However, this was not the case as, not only did all combinations result in at most an overall additive effect (Figure 10), but there was also no significant increase in caspase-3/7 activity (Figure 11A,B). The only exception to this was for RG7388 (4 × GI_50_) compared to RG7388 (4 × GI_50_) + Venetoclax (10 µM). These results are in contrast to Mukherjee et al., who showed that cell death was achieved via apoptosis in a range of melanoma cell lines by combining Venetoclax and S63845 (MCL-1 inhibitor) [56]. The dual targeting of BCL2 (Venetoclax) and MCL-1 (S63845) was also suggested as an effective treatment for patients with myeloma, as both had little effect as single agents but, in combination at low doses, they were able to significantly increase levels of apoptosis. This was also demonstrated for a primary sample taken from a Venetoclax resistant patient. Mechanistically, the combination induced apoptosis in a BAX/BAK-dependent manner, with BCLX_L_ as the major resisting factor [57]. From the studies mentioned above, a triple combination of MDM2 inhibitor with Venetoclax and MIM1 would be worth exploring.

The failures of RG7388 and HDM201 as single agents to induce apoptosis was not due to an inability to stabilise p53 and activate downstream transcriptional targets; therefore, further research is warranted, both to elucidate the mechanistic reasons as to why, and to also explore other avenues. As of now, apart from p53 status, there are no putative biomarkers of response to MDM2 inhibitors. Jeay et al. [58] suggested gene signatures; however, these were later found to be unreliable when taking into consideration cell lines harbouring alterations that inactivated p53 [59]. Clearly, more research is needed to find reliable biomarkers which can be used as predictors of response for both MDM2 and WIP1 inhibitors.

## 5. Conclusions

WIP1 (PPM1D) has a significant anti-apoptotic effect on uLMS, whereas BCL2 or MCL-1 appear to play little or no role in this cancer cell type. Therefore, this research presents the possibility of a combination treatment with MDM2 and WIP1 inhibitors as a potential treatment option for p53^WT^ uLMS that warrants further investigation and the further development of WIP1 inhibitors suitable for in vivo and clinical evaluation.

## Data Availability

The data presented in this study are available on request from the corresponding authors.

## Supplementary Materials

The following are available online at https://www.mdpi.com/article/10.3390/cancers14010014/s1, Figure S1: Original western blots for Figure 4, Figure S2: Original western blots for Figure 8.

## Appendix A

**Table A1:** Details of antibodies used for Western immunoblotting. BSA, Bovine serum albumin.

Protein	Product	Source	Company	Dilution	Diluent
GAPDH	(14C10) #2118	Rabbit	Cell Signalling Technologies	1:1000	Milk
PARP-1	(C2-10) #4338-MC	Mouse	Trevigen	1:1000	Milk
Cleaved-PARP	(Asp 214) #9541	Rabbit	Cell Signalling Technologies	1:1000	Milk
MDM2	OP46	Mouse	EMD Millipore Corporation	1:300	Milk
WIP1	(F-10) sc-376257	Mouse	Santa Cruz	1:1000	Milk
p53	(DO-1) sc-126	Mouse	Santa Cruz	1:500	Milk
pp53	(E9Y4U) #82530	Rabbit	Cell Signalling Technologies	1:1000	BSA
p21	(EA10) OP64	Mouse	EMD Millipore Corporation	1:100	Milk
PUMA	(D30C10) #12450	Rabbit	Cell Signalling Technologies	1:1000	BSA
Cleaved-caspase-3	(Asp175) (5A1E) #9664	Rabbit	Cell Signalling Technologies	1:1000	Milk

**Table A2:** Details of primers used for qRT-PCR.

Gene Name	Primer Sequence
*GAPDH*	F-5′-CAATGACCCCTTCATTGACC-3′ R-5′-GATCTCGCTCCTGGAAGAT-3′
*MCL-1*	F-5′-GTGCCTTTGTGGCTAAACACT-3′ R-5′-AGTCCCGTTTTGTCCTTACGA-3′
*BCL2*	F-5′-GGTGGGGTCATGTGTGTGG-3′ R-5′-CGGTTCAGGTACTCAGTCATCC-3′
*TP53*	F-5′-CAGCACATGACGGAGGTTGT-3′ R-5′-TCATCCAAATACTCCACACGC-3′
*P21*	F-5′-TGTCCGTCAGAACCCATGC-3′ R-5′-AAAGTCGAAGTTCCATCGCTC-3′
*MDM2*	F-5′-AGTAGCAGTGAATCTACAGGGA-3′ R-5′-CTGATCCAACCAATCACCTGAAT-3′
*PUMA*	F-5′-ACCTCAACGCACAGTACGA-3′ R-5′-CTGGGTAAGGGCAGGAGTC-3′
*FAS*	F-5′-AGATTGTGTGATGAAGGACATGG-3′ R-5′-TGTTGCTGGTGAGTGTGCATT-3′
*NOXA*	F-5′-TGCTACACAATGTGGCGTC-3′ R-5′-ACTTGGACATGGCCTCCCTTA-3′
*BCL2L11*	F-5′-TAAGTTCTGAGTGTGACCGAGA-3′ R-5′-GCTCTGTCTGTAGGGAGGTAGG-3′
*TP53INP1*	F-5′-TCTTGAGTGCTTGGCTGATACA-3′ R-5′-GGTGGGGTGATAAACCAGCTC-3′
*BID*	F-5′-ATGGACCGTAGCATCCCTCC-3′ R-5′-GTAGGTGCGTAGGTTCTGGT-3′
*BAX*	F-5′-CCCGAGAGGTCTTTTTCCGAG-3′ R-5′-CCAGCCCATGATGGTTCTGAT-3′
*PPM1D*	F-5′-TTGTCAGAGCTGTGGAGGTG-3′ R-5′-AGTGAGTCGAGGTCGTTTCC-3′

**Table A3:** All statistically significant fold changes in mRNA expression of selective p53 transcriptional targets, as determined by a two-way ANOVA with Tukey′s post hoc test for multiple comparisons between MES-SA cells when treated with RG7388/HDM201 inhibitors and GSK2830371 as single agents or in combination for 6 h.

MES-SA	Treatment	*p*-Value
*CDKN1A*	DMSO vs. 2.5 µM GSK2830371	<0.0001
	DMSO vs. 1 µM RG7388	<0.0001
	DMSO vs. 1 µM HDM201	<0.0001
	DMSO vs. 1 µM HDM201 + 2.5 µM GSK2830371	<0.0001
	DMSO vs. 1 µM RG7388 + 2.5 µM GSK2830371	<0.0001
	2.5 µM GSK2830371 vs. 1 µM RG7388	<0.0001
	2.5 µM GSK2830371 vs. 1 µM HDM201	<0.0001
	2.5 µM GSK2830371 vs. 1 µM HDM201 + 2.5 µM GSK2830371	<0.0001
	2.5 µM GSK2830371 vs. 1 µM RG7388 + 2.5 µM GSK2830371	<0.0001
	1 µM RG7388 vs. 1 µM HDM201	<0.0001
	1 µM RG7388 vs. 1 µM HDM201 + 2.5 µM GSK2830371	<0.0001
	1 µM RG7388 vs. 1 µM RG7388 + 2.5 µM GSK2830371	<0.0001
	1 µM HDM201 vs. 1 µM HDM201 + 2.5 µM GSK2830371	<0.0001
	1 µM HDM201 vs. 1 µM RG7388 + 2.5 µM GSK2830371	<0.0001
	1 µM HDM201 + 2.5 µM GSK2830371 vs. 1 µM RG7388 + 2.5 µM GSK2830371	<0.0001
*MDM2*	DMSO vs. 2.5 µM GSK2830371	0.0088
	DMSO vs. 1 µM RG7388	<0.0001
	DMSO vs. 1 µM HDM201	<0.0001
	DMSO vs. 1 µM HDM201 + 2.5 µM GSK2830371	<0.0001
	DMSO vs. 1 µM RG7388 + 2.5 µM GSK2830371	<0.0001
	2.5 µM GSK2830371 vs. 1 µM RG7388	<0.0001
	2.5 µM GSK2830371 vs. 1 µM HDM201	<0.0001
	2.5 µM GSK2830371 vs. 1 µM HDM201 + 2.5 µM GSK2830371	<0.0001
	2.5 µM GSK2830371 vs. 1 µM RG7388 + 2.5 µM GSK2830371	<0.0001
	1 µM RG7388 vs. 1 µM HDM201	<0.0001
	1 µM RG7388 vs. 1 µM HDM201 + 2.5 µM GSK2830371	<0.0001
	1 µM RG7388 vs. 1 µM RG7388 + 2.5 µM GSK2830371	<0.0001
	1 µM HDM201 vs. 1 µM HDM201 + 2.5 µM GSK2830371	<0.0001
	1 µM HDM201 vs. 1 µM RG7388 + 2.5 µM GSK2830371	<0.0001
*MCL-1*	DMSO vs. 1 µM RG7388 + 2.5 µM GSK2830371	0.0060
*PUMA*	DMSO vs. 1 µM RG7388	<0.0001
	DMSO vs. 1 µM HDM201	<0.0001
	DMSO vs. 1 µM HDM201 + 2.5 µM GSK2830371	<0.0001
	DMSO vs. 1 µM RG7388 + 2.5 µM GSK2830371	<0.0001
	2.5 µM GSK2830371 vs. 1 µM RG7388	<0.0001
	2.5 µM GSK2830371 vs. 1 µM HDM201	<0.0001
	2.5 µM GSK2830371 vs. 1 µM HDM201 + 2.5 µM GSK2830371	<0.0001
	2.5 µM GSK2830371 vs. 1 µM RG7388 + 2.5 µM GSK2830371	<0.0001
	1 µM RG7388 vs. 1 µM HDM201 + 2.5 µM GSK2830371	0.0008
	1 µM RG7388 vs. 1 µM RG7388 + 2.5 µM GSK2830371	<0.0001
	1 µM HDM201 vs. 1 µM RG7388 + 2.5 µM GSK2830371	0.0018
*FAS*	DMSO vs. 2.5 µM GSK2830371	<0.0001
	DMSO vs. 1 µM RG7388	<0.0001
	DMSO vs. 1 µM HDM201	<0.0001
	DMSO vs. 1 µM HDM201 + 2.5 µM GSK2830371	<0.0001
	DMSO vs. 1 µM RG7388 + 2.5 µM GSK2830371	<0.0001
	2.5 µM GSK2830371 vs. 1 µM RG7388	<0.0001
	2.5 µM GSK2830371 vs. 1 µM HDM201	<0.0001
	2.5 µM GSK2830371 vs. 1 µM HDM201 + 2.5 µM GSK2830371	<0.0001
	2.5 µM GSK2830371 vs. 1 µM RG7388 + 2.5 µM GSK2830371	<0.0001
	1 µM RG7388 vs. 1 µM HDM201 + 2.5 µM GSK2830371	<0.0001
	1 µM RG7388 vs. 1 µM RG7388 + 2.5 µM GSK2830371	<0.0001
	1 µM HDM201 vs. 1 µM HDM201 + 2.5 µM GSK2830371	<0.0001
	1 µM HDM201 vs. 1 µM RG7388 + 2.5 µM GSK2830371	<0.0001
	1 µM HDM201 + 2.5 µM GSK2830371 vs. 1 µM RG7388 + 2.5 µM GSK2830371	<0.0001
*NOXA*	DMSO vs. 2.5 µM GSK2830371	<0.0001
	DMSO vs. 1 µM RG7388	<0.0001
	DMSO vs. 1 µM HDM201	<0.0001
	DMSO vs. 1 µM HDM201 + 2.5 µM GSK2830371	<0.0001
	DMSO vs. 1 µM RG7388 + 2.5 µM GSK2830371	<0.0001
	2.5 µM GSK2830371 vs. 1 µM HDM201 + 2.5 µM GSK2830371	<0.0001
	2.5 µM GSK2830371 vs. 1 µM RG7388 + 2.5 µM GSK2830371	<0.0001
	1 µM RG7388 vs. 1 µM HDM201 + 2.5 µM GSK2830371	<0.0001
	1 µM RG7388 vs. 1 µM RG7388 + 2.5 µM GSK2830371	0.0001
	1 µM HDM201 vs. 1 µM HDM201 + 2.5 µM GSK2830371	<0.0001
	1 µM HDM201 vs. 1 µM RG7388 + 2.5 µM GSK2830371	<0.0001
	1 µM HDM201 + 2.5 µM GSK2830371 vs. 1 µM RG7388 + 2.5 µM GSK2830371	<0.0001
*TP53INP1*	DMSO vs. 2.5 µM GSK2830371	<0.0001
	DMSO vs. 1 µM RG7388	<0.0001
	DMSO vs. 1 µM HDM201	<0.0001
	DMSO vs. 1 µM HDM201 + 2.5 µM GSK2830371	<0.0001
	DMSO vs. 1 µM RG7388 + 2.5 µM GSK2830371	<0.0001
	2.5 µM GSK2830371 vs. 1 µM HDM201	<0.0001
	2.5 µM GSK2830371 vs. 1 µM HDM201 + 2.5 µM GSK2830371	<0.0001
	2.5 µM GSK2830371 vs. 1 µM RG7388 + 2.5 µM GSK2830371	<0.0001
	1 µM RG7388 vs. 1 µM HDM201	<0.0001
	1 µM RG7388 vs. 1 µM HDM201 + 2.5 µM GSK2830371	<0.0001
	1 µM RG7388 vs. 1 µM RG7388 + 2.5 µM GSK2830371	<0.0001
	1 µM HDM201 vs. 1 µM HDM201 + 2.5 µM GSK2830371	<0.0001
	1 µM HDM201 vs. 1 µM RG7388 + 2.5 µM GSK2830371	<0.0001
*BID*	DMSO vs. 2.5 µM GSK2830371	0.0242
	DMSO vs. 1 µM RG7388	0.0119
	DMSO vs. 1 µM HDM201 + 2.5 µM GSK2830371	0.0220
	DMSO vs. 1 µM RG7388 + 2.5 µM GSK2830371	0.0039
*BAX*	DMSO vs. 1 µM HDM201	0.0013
	DMSO vs. 1 µM HDM201 + 2.5 µM GSK2830371	0.0001
	DMSO vs. 1 µM RG7388 + 2.5 µM GSK2830371	0.0002
*PPM1D*	DMSO vs. 1 µM RG7388	<0.0001
	DMSO vs. 1 µM HDM201	<0.0001
	DMSO vs. 1 µM HDM201 + 2.5 µM GSK2830371	0.0020
	DMSO vs. 1 µM RG7388 + 2.5 µM GSK2830371	<0.0001
	2.5 µM GSK2830371 vs. 1 µM RG7388	0.0002
	2.5 µM GSK2830371 vs. 1 µM HDM201	<0.0001
	2.5 µM GSK2830371 vs. 1 µM HDM201 + 2.5 µM GSK2830371	0.0088
	2.5 µM GSK2830371 vs. 1 µM RG7388 + 2.5 µM GSK2830371	<0.0001
	1 µM HDM201 vs. 1 µM HDM201 + 2.5 µM GSK2830371	0.0019

**Table A4:** All statistically significant fold changes in mRNA expression of selective p53 transcriptional targets, as determined by a two-way ANOVA with Tukey′s post hoc test for multiple comparisons between SK-UT-1 cells when treated with RG7388/HDM201 inhibitors and GSK2830371 as single agents or in combination for 6 h.

SK-UT-1	Treatment	*p*-Value
*CDKN1A*	2.5 µM GSK2830371 vs. 1 µM RG7388	0.0005
	2.5 µM GSK2830371 vs. 1 µM HDM201	0.0022
	1 µM RG7388 vs. 1 µM RG7388 + 2.5 µM GSK2830371	0.0114
	1 µM HDM201 vs. 1 µM RG7388 + 2.5 µM GSK2830371	0.0381
*MCL-1*	DMSO vs. 1 µM RG7388	0.0006
	2.5 µM GSK2830371 vs. 1 µM RG7388	0.0053
	1 µM RG7388 vs. 1 µM HDM201	0.0085
	1 µM RG7388 vs. 1 µM HDM201 + 2.5 µM GSK2830371	<0.0001
	1 µM RG7388 vs. 1 µM RG7388 + 2.5 µM GSK2830371	0.0300
*NOXA*	DMSO vs. 2.5 µM GSK2830371	<0.0001
	DMSO vs. 1 µM HDM201	<0.0001
	DMSO vs. 1 µM HDM201 + 2.5 µM GSK2830371	0.0280
	2.5 µM GSK2830371 vs. 1 µM RG7388	0.0492
	1 µM RG7388 vs. 1 µM HDM201	0.0017
	1 µM HDM201 vs. 1 µM HDM201 + 2.5 µM GSK2830371	0.0228
	1 µM HDM201 vs. 1 µM RG7388 + 2.5 µM GSK2830371	0.0024
*BCL2L11*	DMSO vs. 2.5 µM GSK2830371	0.0462
	DMSO vs. 1 µM RG7388 + 2.5 µM GSK2830371	0.0136
*TP53INP1*	DMSO vs. 1 µM HDM201	<0.0001
	DMSO vs. 1 µM RG7388 + 2.5 µM GSK2830371	0.0014
	1 µM RG7388 vs. 1 µM HDM201	0.0002
	1 µM RG7388 vs. 1 µM RG7388 + 2.5 µM GSK2830371	0.0058
*BAX*	DMSO vs. 1 µM HDM201	0.0453
