# Supplementary material for: Tipping Growth Inhibition into Apoptosis by Combining Treatment with MDM2 and WIP1 Inhibitors in p53WT Uterine Leiomyosarcoma
Source: Cancers (Basel). 2021 Dec 21;14(1):14. doi: 10.3390/cancers14010014 (PMC8750798; doi:10.3390/cancers14010014)
Supplement: Supplementary file 1 [file cancers-14-00014-s001.zip › cancers-1466336-supplementary.pdf]

Supplementary Materials: Tipping Growth Inhibition into Apoptosis by Combining Treatment with MDM2 and WIP1 Inhibitors in p53^{WT} Uterine Leiomyosarcoma

Victoria Chamberlain, Yvette Drew and John Lunec

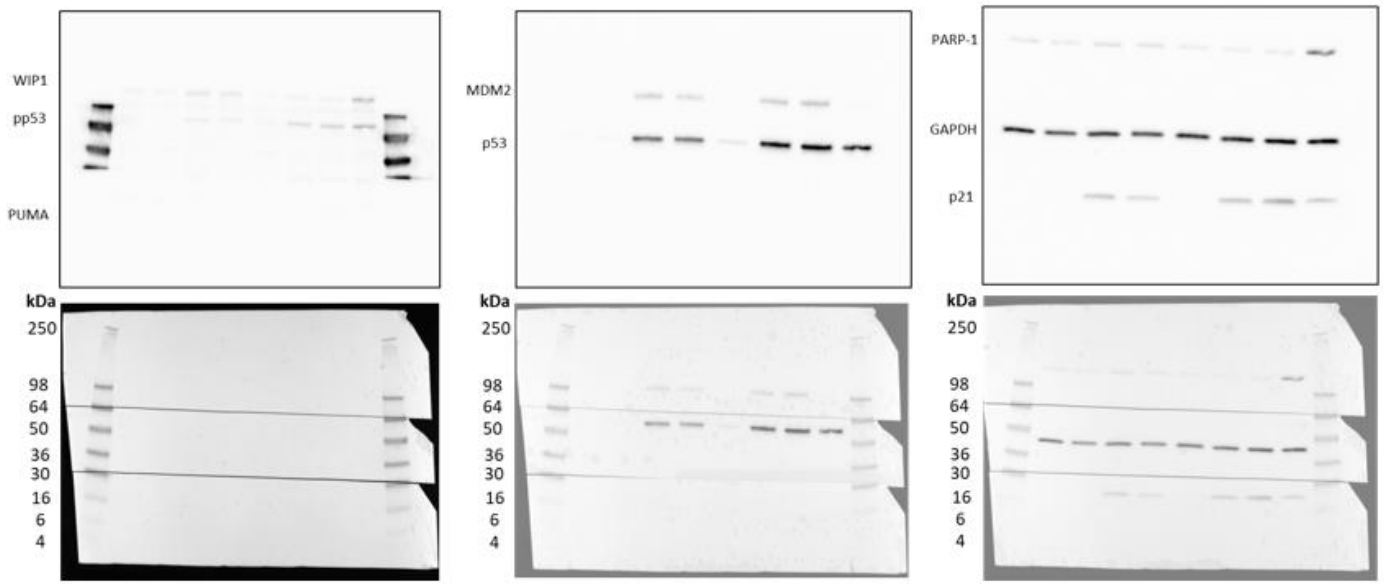

Figure S1. Original western blots for Figure 4.

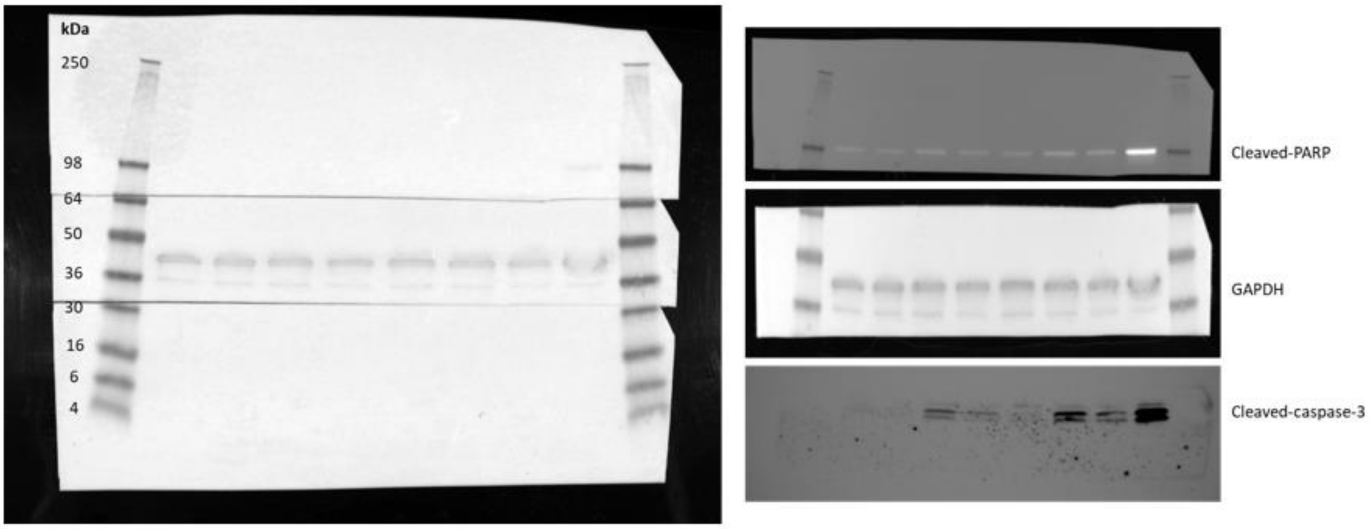

Figure S2. Original western blots for Figure 8.
